# Why Do Marijuana and Synthetic Cannabimimetics Induce Acute Myocardial Infarction in Healthy Young People?

**DOI:** 10.3390/cells11071142

**Published:** 2022-03-28

**Authors:** Jolanta Weresa, Anna Pędzińska-Betiuk, Krzysztof Mińczuk, Barbara Malinowska, Eberhard Schlicker

**Affiliations:** 1 Department of Experimental Physiology and Pathophysiology, Medical University of Białystok, 15-222 Białystok, Poland; jolanta.weresa@umb.edu.pl (J.W.); anna.pedzinska-betiuk@umb.edu.pl (A.P.-B.); krzysztof.minczuk@umb.edu.pl (K.M.); 2 Department of Pharmacology and Toxicology, University of Bonn, 53127 Bonn, Germany

**Keywords:** cannabinoids, cannabinoid receptor, heart, marijuana, myocardial infarction, tachycardia, THC, oxygen consumption, sympathetic system, thrombus

## Abstract

The use of cannabis preparations has steadily increased. Although cannabis was traditionally assumed to only have mild vegetative side effects, it has become evident in recent years that severe cardiovascular complications can occur. Cannabis use has recently even been added to the risk factors for myocardial infarction. This review is dedicated to pathogenetic factors contributing to cannabis-related myocardial infarction. Tachycardia is highly important in this respect, and we provide evidence that activation of CB_1_ receptors in brain regions important for cardiovascular regulation and of presynaptic CB_1_ receptors on sympathetic and/or parasympathetic nerve fibers are involved. The prototypical factors for myocardial infarction, i.e., thrombus formation and coronary constriction, have also been considered, but there is little evidence that they play a decisive role. On the other hand, an increase in the formation of carboxyhemoglobin, impaired mitochondrial respiration, cardiotoxic reactions and tachyarrhythmias associated with the increased sympathetic tone are factors possibly intensifying myocardial infarction. A particularly important factor is that cannabis use is frequently accompanied by tobacco smoking. In conclusion, additional research is warranted to decipher the mechanisms involved, since cannabis use is being legalized increasingly and Δ^9^-tetrahydrocannabinol and its synthetic analogue nabilone are indicated for the treatment of various disease states.

## 1. Introduction

Cannabis is derived from leaves, stems or flowers of the *Cannabis sativa* and *Cannabis indica* plants, which have been known since ancient times, and like other primeval plants, e.g., opium poppy and ephedra, have been used over centuries and are still prominent today [1,2,3]. Mostly, dried flowers or leaves (marijuana) are inhaled through smoking or vaping, but edible products such as cookies, gummies or chocolate are used as well. Cannabis plants contain more than 400 different compounds and about 100 cannabinoids [4]; the latter ones, mainly Δ^9^-tetrahydrocannabinol (THC; previous name Δ^1^-tetrahydrocannabinol) are responsible for the biological effects of cannabis including euphoria, relaxation and changes in perceptions but also dysphoria, anxiety or psychotic symptoms. THC and the nonintoxicating cannabidiol (CBD) are the best-studied cannabinoids [4,5].

**Figure 1:**
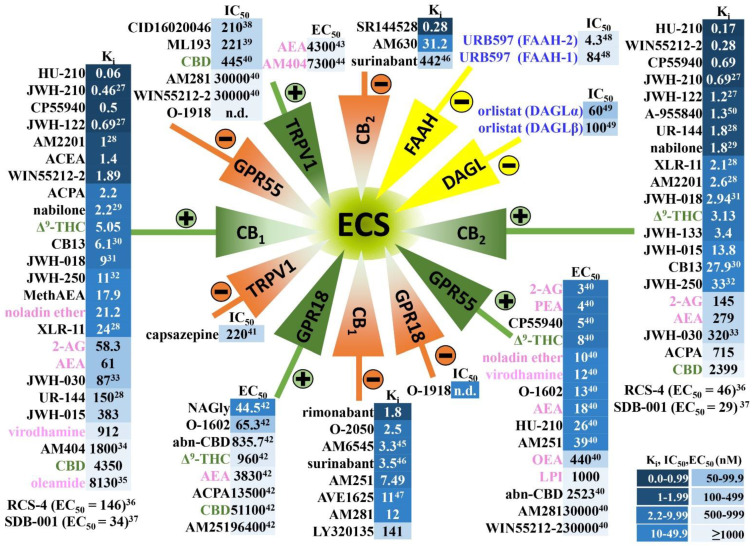
Cannabinoids and their affinities to the classical cannabinoid CB_1_ and CB_2_ receptors and to other receptors sensitive to cannabinoids, as well as to inhibitors of enzymes involved in the synthesis and/or degradation of AEA and 2-AG. Note that the numbers in the superscript indicate the appropriate reference [15,16,17,18,19,20,21,22,23,24,25,26,27,28,29,30,31,32,33,34,35,36,37,38]. The figure presents only phytocannabinoids (green font), synthetic cannabinoids and other compounds discussed in this article (black font), endogenous cannabinoids (pink font) and inhibitors of the endocannabinoid synthesis and degradation (blue font) that have been considered in this review. ECS, endocannabinoid system; the “plus sign” indicates agonism and the “minus sign” antagonism, inverse agonism or inhibition versus the respective receptors/enzymes. The intensity of blue color next to the compound is higher the lower the values of K_i_, IC_50_ or EC_50_ are (expressed in nM). Based on Pertwee et al. [39] unless stated otherwise (superscript). WIN55212-3, inactive S(–)enantiomer of WIN55212-2 [40]; AM404, an inhibitor of anandamide transport [41]. Abbreviations: Δ^9^-THC, Δ^9^-tetrahydrocannabinol; 2-AG, 2-arachidonoylglycerol; abn-CBD, abnormal cannabidiol; ACEA, arachidonoyl-2’-chlorethylamide; ACPA, arachidonylcyclopropylamide; AEA, anandamide; CB_1_, cannabinoid CB_1_ receptor; CB_2_, cannabinoid CB_2_ receptor; CBD, cannabidiol; DAGL, diacylglycerol lipase; ECS, endocannabinoid system; FAAH, fatty-acid amide hydrolase; GPR18, G protein-coupled receptor 18; GPR55, G protein-coupled receptor 55; LPI, L-alpha-lysophosphatidylinositol; MethAEA, methanandamide; n.d., not determined; OEA, oleoylethanolamide; PEA, palmitoylethanolamide; TRPV1 transient receptor-potential cation-channel subfamily V member 1; URB597, inhibitor of fatty-acid amide hydrolase.

THC, CBD, its mixture and the synthetic cannabinoid nabilone are also available in purified or pure form and are used for medical purposes. Cesamet^®^ (nabilone) and Marinol^®^ (THC; IUPAC name: (–)-Δ^9^-trans-tetrahydrocannabinol; INN: dronabinol) are approved for anorexia and weight loss in HIV infection and for nausea and vomiting in cancer chemotherapy; Epidiolex^®^ (cannabidiol) is indicated for Lennox–Gastaut and Dravet syndrome and Sativex^®^ (mixture of CBD + THC, nabiximols) for neuropathic pain from multiple sclerosis and intractable cancer pain [6,7]. Synthetic agonists of cannabinoid receptors (CB-Rs), so-called synthetic cannabimimetics, are lipid-soluble compounds which consist of 20–26 carbon atoms; they volatilize readily during smoking and are undetectable with common drug-screening tests. Nowadays, they are manufactured in illegal laboratories under various brand names such as K2 or Spice and became popular for recreational purposes due to their increased availability in the market [8,9]. Their distribution is not controlled and they are easily available as “seemingly innocent, herbal products”. They possess structural and biochemical similarities and exert even more potent and deleterious adverse effects in comparison to THC due to their greater affinity for the CB_1_-R, the CB-R subtype responsible for the psychotropic effects of cannabis (for affinities of THC and other cannabinoids considered in this review, see Figure 1) [10,11,12,13,14].

A significant increase in global intake of both plant and synthetic cannabinoids has been observed in recent decades. In 2016, 192.2 million people aged 15–64 years used cannabis for nonmedical purposes across the globe (INCB 2018). The underlying reasons of this effect are growing legalization (e.g., in U.S. the number of states legalizing marijuana for recreational purposes is still rising [42]); the impact of mass culture with the common occurrence of marijuana and its symbols in everyday products; and recently, the overall increase in the use of psychoactive substances during the lockdown of the COVID-19 pandemic [43,44,45]. Moreover, in 2016 the WHO reported a dramatic (about tenfold) increase in the THC content of marijuana [11]. 

In an early review about the cardiovascular safety of THC [46], the conclusion was reached that harmful effects occur in people with pre-existing heart disease only. In more recent studies, acute exposure even of young healthy people to cannabis was reported to lead to severe cardiovascular events including myocardial infarction (MI), sudden cardiac death, cardiomyopathy, transient ischemic attack and stroke (e.g., [47,48]. For example, Bachs and Mørland [49] reported six cases of cardiac death in young adults in which THC was present in postmortem blood samples. One of the first convincing studies suggesting that marijuana acts as a trigger for myocardial infarction (MI) was performed by Mittleman et al. [50] who showed that marijuana smokers had a 4.8-fold increased risk of developing MI in the first hour after cannabis exposure. Moreover, a French study reported that cardiovascular disorders, including MI and fatal stroke, were observed among 9.5% of 200 cannabis-related hospitalizations [51]. Therefore, it was recommended that individuals with pre-existing cardiovascular conditions should avoid cannabis [48]. 

In the past five years, cannabis use has been listed among the risk factors of MI (also associated with an increased risk of cardiovascular mortality) in younger patients [52,53] and at least twelve reviews have appeared drawing attention to adverse cardiac effects of cannabinoids (Table 1). They are based on numerous case reports, epidemiologic or retrospective cohort studies encompassing millions of cannabis users, patients or hospitalizations. Their authors conclude that (1) cannabis use is an independent predictor of MI, heart failure and cerebrovascular accidents; (2) young cannabis users are more at risk with respect to hospitalizations for acute MI, arrhythmia and stroke; (3) medical cannabis authorization was associated with an increased risk of visits at emergency departments or hospitalizations for cardiovascular events including stroke and acute coronary syndrome; (4) screening for marijuana use should be performed in young patients with cardiovascular disease; and (5) the increasing risk of MI and other acute cardiovascular events among young cannabis users strongly needs further studies (including clinical trials) to assess cannabis-related cardiovascular implications and to determine the detailed pathophysiology of cardiac adverse events of cannabis (Table 1).

The aims of this review are (1) to give a short introduction of the endocannabinoid system, including cannabinoid receptors and their distribution in areas relevant for cardiovascular effects; and (2) to discuss sites and mechanisms responsible for tachycardia associated with cannabis use in humans. Moreover, we are going to assess whether cannabis use affects (3) the two prototypical pathogenetic mechanisms involved in the development of MI, i.e., thrombus formation and coronary constriction; and (4) further mechanisms which may worsen MI, such as a decrease in energy supply, an increase in energy demand, as well as proarrhythmogenic and cardiotoxic effects.

## 2. Endocannabinoid System in Humans and in Experimental Animals—Components and Anatomical Distribution

The main components of the endocannabinoid system (ECS) are shown in Figure 1. It consists of (i) the endogenous cannabinoids (endocannabinoids) such as anandamide (AEA), 2-arachidonoylglycerol (2-AG), noladin ether, virodhamine, oleamide or endocannabinoid-like compounds such as palmitoylethanolamide (PEA) or oleoylethanolamide (OEA), (ii) their receptors and (iii) the enzymes involved in their synthesis and degradation [63,64]. Cannabinoids act via two main types of cannabinoid receptors, CB_1_-R and CB_2_-R, that belong to the G protein-coupled receptor (GPCR) superfamily. Other targets of endocannabinoids are the orphan G-protein-coupled receptors GPR18 and GPR55. GPR18, which shares low sequence homology with the cannabinoid receptors, is also activated by endogenous L-α-lysophosphatidylinositol (LPI; [65]). Another important cannabinoid-sensitive receptor, which, does not, however, belong to the GPCR superfamily, is the transient receptor-potential vanilloid-type 1 (TRPV1). AEA is among others synthesized by the N-acyl-phosphatidylethanolamine (NAPE) phospholipase D. For 2-AG synthesis, two isoforms of diacylglycerol lipase (DAGL), α and β, are necessary. Fatty-acid amide hydrolases (isoforms FAAH-1 and FAAH-2) and monoacylglycerol lipase (MAGL) are responsible for degradation of AEA and 2-AG, respectively. For removal of AEA, an AEA transporter may also be relevant [64,66].

With respect to THC, two extreme differences to the ECS have to be considered. First, THC has an affinity for CB_1_, CB_2_, GPR18 and GPR55 receptors only (without binding to e.g., TRPV1 receptors; Figure 1). Second, degradation of THC does not involve enzymes such as FAAH or MAGL. For this reason, data with TRPV1 receptors will not be considered in this article at all and results with FAAH and MAGL inhibitors will be discussed only if experiments with appropriate antagonists have revealed that CB_1_, CB_2_, GPR18 and GPR55 receptors are involved. 

To compare acute cardiac functional effects (and their potential mechanisms) in human and in experimental animals we have concentrated on the comparison of the distribution of CB-Rs in the heart, coronary artery, platelets and in brain regions involved in cardiovascular regulation. In the human heart, gene and/or protein expression of CB_1_-Rs and CB_2_-Rs occurs in the left ventricle [67,68,69], right atrium [70] and epicardial adipose tissue [71]. CB_1_-Rs and CB_2_-Rs were also identified in hearts of the guinea pig [72], rat [73,74,75], and mouse [76,77]. In detail, these receptors occur in the left ventricle [78,79] and left atrium of the rat [80], and in the left ventricle of the dog [81] and the mouse [67,82,83]. 

The occurrence of the GPR55 was described in human [84] and mouse [85] hearts, in rat neonatal cardiomyocytes [86] and left ventricles [79] and in mouse ventricles [87]. The GPR18 occurs in rat left ventricles [79] and in rat fetal cardiac tissue, but not the maternal heart [88].

Receptors sensitive to cannabinoids were also found in coronary arteries of humans (CB_1_-Rs, CB_2_-Rs; [89]) and rats (CB_1_-Rs; [90]). In human platelets, the occurrence of both classical CB-Rs [91] and the GPR55 [84] was shown.

CB_1_ receptors were detected in high densities in many brain regions of humans and experimental animals [92]. They also occur in brain regions involved in cardiovascular regulation, including the rostral ventrolateral medulla (RVLM; [93,94]), the bed nucleus of the stria terminalis (BNST; [95,96]), the ventral medial prefrontal cortex (vMPFC; [97]), the paraventricular nucleus of the hypothalamus (PVN; [98]), the nucleus tractus solitarii (NTS; [99]) and the dorsal periaqueductal gray (dPAG; [97,100]).

Receptors sensitive to cannabinoids have so far not been identified in hearts of rhesus monkeys, cats and rabbits. Moreover, the components of the ECS have not been detected in the cardiac conduction system of humans or experimental animals. To the best of our knowledge, GPR18 has not been identified in human cardiac tissue. 

## 3. Tachycardia—General 

Zuurman et al. [101] clearly showed in their review analyzing 165 articles, which are dedicated to the acute effects of cannabis or THC on the central nervous system (CNS) and HR in healthy volunteers and are based on 318 tests (or test variants), that an increase in HR is the most reliable biomarker to study the effects of cannabis. Tachycardia leading to complex adverse cardiac consequences such as a decrease in cardiac-stroke volume or myocardial oxygen supply–demand imbalance is regarded as a potent predictor of cardiovascular morbidity and mortality [102,103]. In this context, it is of interest that a reduction in heart rate (HR) induced by various drugs has a beneficial effect in patients with MI [103].

Table 2 shows that a THC-induced increase in HR occurs after:

- smoking of cigarettes (joints) [104,105,106,107,108,109,110,111,112,113,114];
- inhalation by vaporizer [106,111,115,116,117,118];
- peroral intake [111,119,120] (the synthetic form of THC, nabilone, has to be listed here as well (Cesamet^®^; [121]);
- intravenous (i.v.) injection [122,123,124,125,126];
- administration of an oromucosal spray containing THC and cannabidiol (Sativex^®^; [127]).

**Table 2:** Cardiac effects of acute cannabinoid administration in humans.

Number (Characterization, Age in Years)	Agonist	Dose (mg) *	Application	Cardiac Effects	Comments/Suggested Mechanisms and Involvement of CB_1_-Rs	References
10 healthy volunteers (21–33)	THC	1–40	cigarette	dose-dependent ↑HR; ↑BP	changes in BP better correlated to HR than to doses; antagonists not studied	[104]
16 healthy volunteers (18–42)	THC	25	cigarette	↑HR; ↓BP: normotensive < hypertensive persons	n.a.	[105]
6 healthy volunteers (18–30)	THC	10	cigarette	↑HR; ↑BP	tachycardia resulting from β-AR activation (diminished by propranolol 120 mg p.o.)	[107]
10 healthy volunteers(30–40)	THC	10	cigarette	↑HR; ECG: ↑amplitude and ↓width of P wave in Lead 2 and inversion of T wave in Lead 3	tachycardia is mediated via β-ARs, since it was prevented by propranolol (40 mg/kg p.o.) but not by atropine (0.6 mg/kg s.c.)	[108]
14 healthy volunteers (20–31)	THC	6	cigarette	↑HR and ↑left ventricular performance (mean rate of internal diameter shortening)	tachycardia not accompanied by ↑plasma NA levels, since the respective maximal increases took place at 10 and 30 min, respectively	[110]
21 experienced users of cannabis (21–45)	THC	20–60	1 to 3 cigarettes	↑HR; ↑cardiac output ↔stroke volume ↔ejection fraction	marijuana has no significant effect on myocardial contractility independent of its effect on HR	[113]
91 cannabis users (19–25); double-blind, placebo-controlled, parallel-group randomized clinical trial	THC	94	cigarette	↑HR; peak HR (~40 beats/min) and plasma THC concentration (~55 ng/mL) at 5 min; ↑HR different until +4 h	n.a.	[112]
42 volunteers (mean age of 29); randomized, double blind, parallel group design	cannabis	2.8% THC	cigarette	↑HR	acute tachycardia depends on CB_1_-Rs since it was diminished by acute rimonabant (90 mg/kg p.o.) or its chronic application (40 mg/kg for 8 and 15 days)	[109]
16 healthy volunteers (mean age of 28)	THC UR-144	1, 1.5 10, 20	joints (smoking)	↑HR, ↑BP ↑HR, ↑BP	(THC and the preferential CB_2_-R agonist UR-144 were administered in joints containing tobacco.)	[114]
16 healthy volunteers (mean age of 30)	JWH-122 JWH-210	1 1.25	joints (smoking)	↑HR, ↑BP ↔HR, ↔BP	(compounds with high potency at CB_1_-/CB_2_-Rs, respectively)	[128]
17 healthy volunteers (mean age of 27)	THC	10 25	smoked or vaporized	↑HR ↑HR	THC-induced tachycardia slightly higher in the case of vaporization	[106]
36 healthy volunteers (18–31)	THC	2, 4 and 6	inhalation by vaporizer	↑HR in a dose-dependent manner	THC-induced tachycardia inhibited by the CB_1_-R antagonist AVE1625 (20, 60, 120 mg p.o.)	[116]
30 healthy volunteers (18–45); double-blind, placebo-controlled, randomized, four-period six-sequence crossover study	THC	2, 4 and 6	inhalation by vaporizer	↑HR	THC-induced tachycardia was inhibited by the CB_1_-R antagonist surinabant 20 and 60 mg p.o.	[118]
12 healthy volunteers (21–27)	THC	2, 4, 6 and 8	vaporized	sharp ↑HR and rapid decline	THC-induced tachycardia is dose-dependent	[115]
12 healthy volunteers	THC	2, 4, 6 and 8	vaporized	↑HR	different sites of action for cardiac and CNS responses suggested: average population equilibration half-life for HR 8 min and for CNS 39–85 min	[117]
11 frequent and 9 occasional cannabis smokers (mean age of 27 and 29, respectively)	THC	~54	smoked vaporized oral	↑HR; ↑carbon monoxide ↑HR ↑HR	smoking produced higher increase in carbon monoxide compared to vaporization	[129]
84 healthy volunteers (mean age of 32), naturalistic, ad libitum use	THC	average 51 average 16	smoked or vaporized (flower cannabis), oral (edible cannabis)	↑HR ↑HR	the flower group started with lower basal HR than the edible group at pre-use but had higher average HR at post-use	[111]
16 healthy volunteers (mean age of 26)	THC CBD	10 600	capsules	↑HR ↔HR	tachycardia induced by THC but not by CBD (has low affinity to CB_1_-Rs)	[119]
14 healthy volunteers (21–45); randomized, double-blind design	nabilone Cesamet^®^ dronabinol Marinol^®^	4, 6, 8 10, 20	capsules capsules	dose-dependent ↑HR, ↓systolic BP ↔HR, ↓systolic BP	nabilone has better bioavailability than dronabinol (THC)	[121]
37 healthy volunteers (18–35)	THC	7.5, 15	capsules	↑HR, ↓heart rate variability, ↔pre-ejection period, ↔BP	tachycardia results from parasympathetic inhibition; no changes in sympathetic tone	[120]
9 healthy volunteers (mean age of 21.4)	THC (Namisol^®^)	6.5 and 8.0	tablets	slight ↑HR (by about 5 beats/min)	n.a.	[130]
11 healthy volunteers (≥65)	THC (Namisol^®^)	3, 5 or 6.5	tablets	no clinically relevant changes in HR and ECG parameters	n.a.	[131]
5 volunteers (22–29); regular marijuana smokers (at least once a day)	THC	30 μg/kg	i.v.	↑HR	tachycardia results from sympathetic stimulation and parasympathetic inhibition (diminished by i.v. propranolol and atropine 0.2 and 0.04 mg/kg, respectively)	[122]
20 healthy male volunteers (22–30)	THC	25 μg/kg ≈ 5 mg in one marijuana cigarette	i.v.	↑HR, ↑total electromechanical systole; ↑left ventricular ejection time; ↓pre-ejection period	THC-induced changes in cardiac performance via the autonomic nervous system: partially diminished by propranolol 0.1 mg/kg i.v. and totally by propranolol 0.1 mg/kg plus atropine 2 mg/kg i.v.	[123,124]
21 healthy volunteers	THC CBD	0.2 mg/min 1.8 mg/min	i.v.	↑HR ↑HR	CBD increases HR only at a much higher dose than THC	[126]
6 patients undergoing diagnostic ECG evaluation	THC	25 μg/kg	i.v.	↓sinus length; ↓mean sinus node recovery, ↓maximal sinus node recovery times; ↓mean calculated sinoatrial conduction; ↓mean A-V nodal effective and functional refractory periods	(25 µg/kg i.v. correspond to ≈5 mg in one marijuana cigarette) enhancement of sinus automaticity and facilitation of sinoatrial and A-V nodal conduction	[125]
9 cannabis users	THC Sativex^®^: THC: CBD	5, 15 16.2:15.0	oromucosal sprays	comparable ↑HR induced by THC and Sativex^®^	CBD fails to diminish the THC-induced tachycardia	[127]

This table does not include individual case reports because of the marked heterogeneity in terms of dose, concomitant diseases or concurrent drugs and stimulants (which, in turn, might lead to adverse cardiac reactions). * if not stated otherwise; A-V, atrioventricular; β-AR, β-adrenergic receptor; BP, blood pressure; CB_1_-R, CB_2_-R, cannabinoid CB_1,_ CB_2_ receptor; CBD, cannabidiol; CNS, central nervous system; ECG, electrocardiography; HR, heart rate; i.v., intravenous(ly); NA, noradrenaline; n.a., not applicable; p.o., per os; s.c., subcutaneous(ly); THC, Δ^9^-tetrahydrocannabinol.

Significant tachycardia also occurs after smoking of UR-144, JWH-122 and JWH-210 [114,128] and other synthetic cannabimimetics (reviewed by Zawilska et al. [8] and Chung et al. [9]). Nonetheless, quantitative or even qualitative differences exist, depending on the route of administration, the compound under consideration and the pharmaceutical formulation. Thus, comparable increases in HR took place 0.5 h after smoking or vaporization but only 1.5 h after oral administration of the same dose of THC [129]. An increase in HR was induced by nabilone (Cesamet^®^) but not by higher doses of THC (Marinol^®^), because of the better bioavailability of nabilone compared to THC [121]. Namisol^®^, another oral formulation of THC, led to only slight and not clinically relevant changes in HR [130,131].

Cannabinoid-induced tachycardia in humans is mediated by cannabinoid CB_1_ receptors (Table 2), since it was antagonized by three different CB_1_-R antagonists including rimonabant [109], AVE1625 [116] and surinabant [118]. Moreover, cannabidiol (CBD), which possesses very low affinity to CB receptors (see Figure 1), failed to modify HR [119] or increased HR only at a much higher dose than THC did [126]. CBD (believed to be an antagonistic principle against some of the effects of THC; [132]) did not modify the tachycardia induced by THC [127].

In order to determine the mechanism and site of action of the tachycardic effect of THC, it is of interest to include experiments on experimental animals since many studies in humans are not possible for ethical reasons. Since THC was less frequently used in experiments on animals than in humans, not only cardiac effects of THC but also of various other CB-R ligands have been considered in Table 3, Table 4 and Table 5. 

Importantly, THC injected i.v. increased HR only in one early publication performed on conscious rhesus monkeys [133]. In two other publications on conscious rhesus monkeys THC given intraperitoneally (i.p.) and intramuscularly (i.m.) induced bradycardia [134,135] probably via CB_1_-Rs (Table 3). The above receptors are probably activated by endogenous cannabinoids or are constitutively active, since rimonabant i.v., which diminished the THC-elicited bradycardia [135], produced tachycardia by itself in conscious rhesus monkeys [136]. THC-induced bradycardia in conscious animals also occurred independent from the route of its administration in other species such as mongrel dogs (i.v., [137]), rats (i.p., [142]) and mice (i.p., [25]). Sativex^®^, administered as spray, had no effect in beagle dogs [138].

How can we explain such a drastic difference in the cardiac effects of THC between humans and experimental animals? Of course, species differences may be responsible. In this context, one should keep in mind that the low resting HR (~70 beats/min) in humans results from strong parasympathetic dominance [165]. By contrast, basal HR in the experimental animals listed in Table 3 were higher than in humans, pointing to differences in the balance between sympathetic and parasympathetic tone. It amounted to (in beats/min) 100–130 [133], ~130 [136], 140–160 [134] and 190–230 [135] in conscious rhesus monkeys, ~90 in mongrel dogs [137], 350–400 in rats [142] and ~400 in mice [25]. Thus, we cannot exclude the possibility that THC (and/or other cannabinoids) increases HR only in the case of a low basal HR such as in humans.

The cardiovascular effects of cannabinoids have also been studied in anaesthetized animals (for review, see Malinowska et al. [166]; Table 3). Regarding changes in HR, THC i.v. increased HR in mongrel dogs anaesthetized with morphine plus chloralose, but reduced it in conscious animals [137]. Tachycardia was also induced by the highest dose of THC (30 mg/kg i.v.) in anaesthetized rats [155]. In other cases, decreases in HR were reported (i) for THC in anaesthetized cats [151,152] and rats [142,155], (ii) for THC and ∆^8^-THC (previous name Δ^6^-THC) and the synthetic cannabinoids WIN55212-2, CP55940, HU-210, JWH-030, JWH-015 and ACPA in anaesthetized rats [152,155,156,157,159] and (iii) for AEA in anaesthetized mice (CB_1_ receptor-mediated Phase II; [158]). The cannabinoid-induced bradycardia in anaesthetized rats, as in conscious animals, is mediated via CB_1_-Rs since these responses were diminished by rimonabant [155,157,159] but not by the CB_2_-R antagonist SR144528 [157].

## 4. Tachycardia—Mechanisms

The site/mechanism of action involved in the tachycardic effect of THC in humans may be

  i. the heart itself;
 ii. the autonomic nervous system;
iii. the central nervous system;
iv. the baroreceptor reflex.

### 4.1. Heart

The possibility that THC elicits tachycardia via activation of CB_1_-Rs in the sinoatrial node is not plausible, since these receptors are G_i/o_ protein-coupled, i.e., inhibitory. The possibility that THC acts via the β_1_-adrenoceptors activated by noradrenaline (NA) can be excluded, since THC is devoid of a sufficient affinity for this type of receptors [149,163].

Another possibility might be that THC affects the β_1_-adrenoceptor-mediated effect of NA via allosteric modulation. Maggo and Ashton [167] found in isolated rat right atria that WIN55212-2 and MethAEA slightly increased the chronotropic effect of NA. The authors suggest the potential involvement of CB_1_-Rs (as opposed to CB_2_-Rs) in this effect, but they did not use any antagonists.

**Table 4:** Effects of cannabinoid-receptor agonists on heart preparations and platelets of humans and experimental animals in vitro.

Species	Isolated Heart Preparation	CB-R Ligands, Concentrations ^1^	Effects	Possible Mechanisms (CB_1_-Rs, CB_2_-Rs, Others) if Determined	References
humans	right atrial muscle	AEA, MethAEA and HU-210 AM251	↓contractility ↑contractility	CB_1_-R-mediated negative inotropic effect of AEA (antagonized by AM251 but not by AM630); endogenous tone at CB_1_-Rs	[70]
	right atrial appendages	AEA and CP	↓ electrically stimulated [^3^H]-NA release	presynaptic inhibitory CB_1_-Rs (effect antagonized by RIM and LY320135)	[168]
Wistar rats	perfused heart ^2^	THC	↑HR, ↓CF, ↓ cardiac activity	cardiotoxicity, antagonists not used	[169]
SD rats	perfused heart ^2^	THC ∆^8^-THC	↑HR, ↓force of contraction ↔HR (arrhythmia), ↓force of contraction	antagonists not used	[170]
Wistar rats	perfused heart ^2^	HU-210	↓HR	antagonists not used	[171]
Wistar rats	perfused heart ^2^	HU-210, RIM and SR144528	↔HR, ↓LVDP, ↓dp/dt max, ↓dp/dt min	negative inotropism, mechanism unclear; partial agonism of RIM and SR144528?	[172]
Wistar rats	perfused heart ^2^	HU-210	↓HR, ↓LVDP, ↓MRC, ↓MRR, ↓LVEDP alone and after ISO (100 nM)	negative chrono- and inotropism (mechanism?); ↓cardiostimulatory effect of ISO	[157,173]
SD rat	perfused heart ^2^	AEA, MethAEA and ACEA PEA and JWH-015	↓LVDP, ↑CF ↔ LVDP, ↔CF	novel sites mediating AEA-induced negative inotropism (reduced by RIM and SR144528 but not AM251, AM630, CAPZ) and coronary vasodilation (reduced by RIM, SR144528 and AM251, but not AM630 and CAPZ)	[174]
Wistar rats	perfused heart ^2^	AEA	↔HR, ↔CF, ↓dp/dt max, ↓LVSP	antagonists not used	[175]
Wistar rats	perfused heart ^2^	oleamide	↑CF	CB_1_-R suggested but no proven	[176]
Wistar rats	perfused heart ^2^ with VP-induced coronary preconstriction	AEA or ACEA JWH-133 THC	↔HR, ↑CF, ↑LVSP ↑CF, ↑LVSP ↓CF, ↓LVSP	coronary vasodilation and positive inotropism of AEA and ACEA (but not of THC and JWH-133) mediated via CB_1_-Rs (reduced by RIM and AM251 but not by SR144528 and O-1918); effects of THC and JWH-133 not modified by AM251 or AM630	[175]
SD rats	perfused heart ^2^	2-AG WIN-2	↑CF, ↔LVDP ↑CF, ↓LVDP	coronary vasodilation is mediated via CB_1_-Rs; diminished by O-2050; negative inotropic effect by WIN-2 but not by 2-AG	[77]
SD rats	perfused heart ^2^	O-2050 and orlistat	↓effects induced by Ang II including ↓LVDP, ↓dp/dt max, ↓dp/dt min and ↓CF	2-AG reduces negative inotropism + coronary constriction of Ang II via CB_1_-Rs	[77]
SD rats	perfused heart ^2^	JWH-030 30 (but not 3) µM	↓LVEDP, ↔LVSP, ↔LVDP, ↔HR	JWH-030 reduces cell viability via CB_2_-Rs (effect of AM630; no effect of RIM)	[156]
SD rats	perfused heart ^2^ or tachypaced	CB13	↔ dp/dt max, dp/dt min, HR, AV interval, LVDP, ↓tachypa- cing-induced shortening of the atrial effective refractory period	no effects on basal hemodynamic properties; beneficial effects against atrial fibrillation (antagonists not used)	[80]
Wistar rats	intramural coronary resistance artery	WIN-2	relaxation	coronary vasodilation mediated by CB_1_-Rs (antagonized by O-2050 and AM251); CB_1_-R blockade enhanced myogenic tone	[90]
guinea pigs	atria	WIN-2 abn-CBD	↓electrically stimulated [^3^H]-NA release ↔ electrically stimulated [^3^H]-NA release	presynaptic CB_1_-R (antagonized by RIM) but no GPR18 on sympathetic nerve endings	[177]
SD rats	atria	AEA, THC, PEA, JWH-015	↓stimulated [^3^H]-NA release	presynaptic CB_1_-Rs on sympathetic nerve endings (antagonism by RIM)	[178]
Wistar rats	right atria	WIN-2, MethAEA JWH-133	↔ HR; ↑chronotropic effect of NA ↔HR; ↔chronotropic effect of NA	slight enhancement of chronotropic effect of NA (antagonists not used)	[167]
Wistar rats	right atria	CP, CBD AM251 or AM630	↔chronotropic effect and ↑inotropic effect of ISO chronotropic effect of ISO ↑ 1 µM; ↓ 3 µM	mechanism of the effect of CP, AM251 and AM630 on the positive inotropism and/or chronotropism of ISO unclear	[179]
Wistar rats	left atria	AEA AEA ACEA JWH-015	↔dF/dt + AM251: ↑dF/dt + AM630: ↓dF/dt ↓dF/dt ↑dF/dt	negative inotropic effect via CB_1_-Rs and positive inotropic effect via CB_2_-Rs (AM251 and AM630 reduced the effects of ACEA and JWH-015, respectively)	[180]
rabbits	left ventricular myocytes	A-955840	↓FS, ↓dL/dt max, ↓L-type calcium current	CB_2_-R agonist A-955840 has negative inotropic effect independent of CB_1_-Rs and CB_2_-Rs (not modified by RIM and SR144528)	[38]
SD rats	ventricular myocytes	CB13	↔ contractile function	no inotropic effect	[18]
mice GPR55^-/-^	left ventricular cardiomyocyte	GPR55 deletion	↑diastole sarcomere length ↔systole sarcomere length ↑transient Ca^2+^ amplitude ↓time from peak contraction to 50% and 90% relaxation	contractile changes dependent of GPR55	[85]
SD rats	homogenized ventricles	THC ∆^8^-THC	↓adenylate cyclase activity ↔adenylate cyclase activity	↓adenylate cyclase activity may lead to cardiac depressant action of THC	[181]
humans	platelets	THC	↑expression of glycoprotein IIb-IIIa and P-selectin involved in platelet activation	antagonists not used	[91]
	platelets	THC	↓adrenaline- or ADP-induced aggregation	antagonists not used	[182]
	platelets	AEA	↑platelet activation ↑intracellular Ca^2+^ concentration	AA metabolites not involved (lack of effect of ASA and an FAAH inhibitor)	[183]
	platelets	AEA	↔aggregation ↓aggregation induced by collagen, ADP, AA and TXA_2_ analogue but not by thrombin	antagonists not used	[184]
	platelets	2-AG	↑aggregation; ↑intracellular Ca^2+^ concentration; ↑AA and TXB_2_ release	CB_1_-Rs involved (inhibition by RIM but not by SR144528 and AA derivatives)	[185]
	platelets	THC 2-AG AM251	↔aggregation ↑aggregation ↔aggregation induced by ADP and thrombin	2-AG induced aggregation was independent from CB_1_- and CB_2_-Rs (not antagonized by AM251 and AM630) but dependent on the conversion to AA (inhibited by MAGL inhibitor and ASA)	[186]
	platelets	2-AG ACEA, JWH-015	↑aggregation, platelet activation ↔aggregation	platelet aggregation induced by 2-AG is independent from CB_1_- and CB_2_-Rs (not antagonized by RIM or SR144528 but connected with ↑TXA_2_)	[187]
	platelets	2-AG, virodhamine AEA, ACEA, JWH015	↑aggregation, platelet activation ↔aggregation	platelet aggregation induced by 2-AG and virodhamine independent from CB_1_- and CB_2_-Rs but inhibited by MAGL inhibitor, ASA and TXA_2_-R antagonist	[188]
	platelets	AM251 or AM630	↔platelet count ↔aggregation induced by collagen, AA and ADP	platelet aggregation is independent of CB_1_- and CB_2_-Rs	[189]
	platelets	LPI	↔aggregation ↓aggregation induced by ADP	GPR55 involved (effect of LPI reversed by the GPR55 antagonist CID16020046)	[26]
rabbits	platelets	THC	↓aggregation induced by ADP and PAF; ↓5-HT release from platelets	antagonists not used	[182]
rabbits	platelets	AEA HU-210	↑aggregation ↔aggregation	platelet aggregation induced by AEA independent from CB_1_-Rs (not antagonized by RIM but reduced by ASA)	[190]
mice	homogenised hearts	THC 100 µM	↓oxygen consumption	antagonists not used	[191]
mice	cardiac mitochondria	THC 0.1 and 0.2 µM	↓oxygen consumption ↓mitochondria coupled respiration	↓oxygen consumption; not dependent on CB_1_-Rs (similar changes in CB_1_^−/−^ mice)	[192]
beef	cardiacmitochondria	THC 120 µM	↓respiration ↓ oxygen consumption	↓mitochondrial oxygen consumption; antagonists not used	[193]
rats	cardiac mitochondria	THC, HU-210, AEA THC and HU-210	↓oxygen consumption and ↓mitochondrial membrane potential ↑mitochondrial hydrogen peroxide production	↓mitochondrial oxygen consumption; antagonists not used	[194]
Wistar rats	cardiac mitochondria	THC up to 500 µM	↔ROS production, no mitochondrial swelling ↔membrane potential, no oxidative stress, no lipid peroxidation	THC is not directly toxic in isolated cardiac mitochondria, and may even be helpful in reducing mitochondrial toxicity	[195]
SD rats	neonatal ventricular myocytes	CB13	prevents ET1–induced ↓mitochondrial bioenergetics and mitochondrial membrane depolarization	improvement in cardiac mitochondrial function (precise mechanism unclear)	[196]
sheep	Purkinje fibers	THC	↑APD_90_	antagonists not used	[197]
rabbits	Purkinje fibers	JWH-030 JWH-210	↓APD_90_, ↔resting membrane potential ↔APD_90_, ↔resting membrane potential	only the highest concentration of JWH-030 reduces APD (mechanism unclear)	[156]
rabbits	sinoatrial node samples	AEA	↓AP duration and ↓AP amplitude	↓AP duration and ↓AP amplitude in SAN pacemaker cells via CB_1_-Rs (blocked by AM251 but not by AM630)	[198]
Wistar rats	papillary muscles; ventricular myocytes	AEA	↓AP duration ↓AP amplitude ↓L-type Ca^2+^ current	antiarrhythmic properties; ↓AP and ↓L-type Ca^2+^ current through CB_1_-Rs (antagonized by AM251 but not AM630)	[199]
Wistar rats	ventricular myocytes	AEA	↓I_to,_ ↑I_KATP_, ↔I_ss_, ↔I_K1_	antiarrhythmic and cardioprotective properties: ↓I_to_ independent of CB_1_- and CB_2_-Rs; ↑I_KATP_ mediated via CB_2_-Rs (antagonized by AM630 but not AM251)	[200]
Wistar rats	ventricular myocytes	AEA JWH-133	↔NCX1 [Ca^2+^]_i_: normal conditions; ↓NCX1 and [Ca^2+^]_i_: ischemia ↓NCX1 and [Ca^2+^]_i_: ischemia	↓calcium overload through ↓NCX1 during ischemia via CB_2_-Rs (antagonized by AM630 but not by AM251; mimicked by JWH-133)	[201]
SD rats	neonatal ventricular myocytes	AEA, MethAEA, JWH-133 and CB13	↓ET1-induced induction of markers of hypertrophy	antihypertrophic properties via CB_1_- and CB_2_-Rs (antagonism by AM251/AM281 and AM630, respectively)	[18]
SD rats	neonatal ventricular myocytes	*extracellular* LPI administration *intracellular* LPI administration	↑Ca^2+^ entry via L-type Ca^2+^ channels, long-lasting membrane depolarization ↑Ca^2+^ release from endolysosomal Ca^2+^ channels, short-lived membrane hyperpolarization	GPR55 Rs at the sarcolemma: ↑Ca^2+^ entry via L-type Ca^2+^ channels, leading to depolarization; GPR55 Rs at membranes of intracellular organelles: ↑intracellular Ca^2+^ release, leading to hyperpolarization (all effects blocked by ML193)	[86]
guinea pigs	ventricular cardiac nuclei	AEA	↓IP_3_-mediated nuclear Ca^2+^ release	involves CB_1_- and CB_2_-Rs (effect reversed by AM251 and AM630, respectively)	[72]
rat cardiomyoblast cell line	H9c2 cells	THC-OH and THC-COOH THC	↑cell migration and proliferation, ↑cell death and significant deterioration in cellular architecture ↔cell morphology or viability	the key metabolites of THC, as opposed to THC itself, elicit toxic cardiac effects (note that THC does not undergo metabolism in H9c2 cells)	[202]
rat cardiomyoblast cell line	H9c2 cells	JWH-030, JWH-210, JWH-250 or RCS-4	all 0.1–100 µM: ↓cell viability, ↑cell apoptosis	synthetic cannabinoids induce cardiotoxicity via CB_2_-Rs (reduced by AM630 but not RIM)	[156]
mice	HL-1 atrial cardiomyocyte	THC 10 and 30 µM	↑ER stress and apoptosis	cardiotoxicity independent of CB_1_-/CB_2_-Rs (not blocked by AM251 and AM630)	[203]

Unless stated otherwise, antagonists did not modify cardiac parameters. ^1^ concentrations of drugs (µM) usually not given; ^2^ spontaneously beating. ↑increase; ↓decrease; ↔no effect; [^3^H]-NA, [^3^H]-noradrenaline; [Ca^2+^]_i_, intracellular Ca^2+^ concentration; Δ^8^-THC, Δ^8^-tetrahydrocannabinol (formerly Δ^6^-THC); 2-AG, 2-arachidonoylglycerol; 5-HT, 5-hydroxytryptamine; AA, arachidonic acid; abn-CBD, abnormal cannabidiol; ACEA, arachidonoyl-2′-chlorethylamide; ADP, adenosine diphosphate; AEA, anandamide; Ang II, angiotensin II; AP, action potential; APD, action potential duration; APD_90_, action potential duration at 90% repolarization; ASA, acetylsalicylic acid; AV, atrioventricular; CAPZ, capsazepine; CB_1_-R, cannabinoid CB_1_ receptor; CB_2_-R, cannabinoid CB_2_ receptor; CBD, cannabidiol; CF, coronary flow; CP, CP55940; dF/dt, contractility; dL/dt max, maximal shortening velocity; dp/dt max, maximum rates of contraction; dp/dt min, maximum rates of relaxation; ER, endoplasmic reticulum; ET1, endothelin-1; FAAH, fatty-acid amide hydrolase; FS, fractional shortening; GPR18, G protein-coupled receptor 18; GPR55, G protein-coupled receptor 55; HR, heart rate; I_K1_, inward-rectifier potassium current; I_KATP,_ ATP-sensitive potassium current; IP_3_, inositol 1,4,5-trisphosphate receptor; ISO, isoprenaline; I_ss_, steady-state outward potassium current; I_to_, transient outward potassium current; LPI, L-α-lysophosphatidylinositol; LVDP, left ventricular developed pressure; LVEDP, left ventricular end-diastolic pressure; LVSP, left ventricular systolic pressure; MAGL, monoacylglycerol lipase; MethAEA, methanandamide; MRC, maximum rate of contraction; MRR, maximum rate of relaxation; NA, noradrenaline; NCX1, Na^+^/Ca^2+^exchanger; PAF, platelet-activating factor; PEA, palmitoylethanolamide; RIM, rimonabant; ROS, reactive oxygen species; Rs, receptors; SAN, sinoatrial node; SD, Sprague Dawley; THC, Δ^9^-tetrahydrocannabinol (formerly Δ^1^-THC); THC-OH, 11-hydroxy-Δ^9^-THC; THC-COOH, 11-nor-9-carboxy-Δ⁹-tetrahydrocannabinol; TXA_2,_ thromboxane A_2;_ TXA_2_-R, thromboxane A_2_ receptor; TXB_2,_ thromboxane B_2_; VP, vasopressin; WIN-2, WIN55,212-2.

In a similar study on rat atria [179], CP55940 (or CBD) did not affect the positive chronotropic effect of isoprenaline, an unselective β-adrenoceptor agonist. By contrast, both AM251 (CB_1_-R antagonist) and AM630 (CB_2_-R antagonist) increased the effect of isoprenaline at 1 µM and decreased it at 3 µM. It must be recalled in this context that a negative and positive inotropic effect occurs following CB_1_- and CB_2_-R activation in the isolated left atrium of the rat, respectively [180]. It is surprising that both antagonists influenced the effect of isoprenaline in an identical concentration-dependent manner in the study of Weresa et al. [179] and it is unclear whether the results obtained for the antagonists are of interest for the effect of THC.

However, THC and/or synthetic cannabinoids failed to modify the tachycardia induced by isoprenaline in spinal dogs [149], pithed rats [40,163] and rabbits [139]. They also failed to affect the bradycardia induced by the muscarinic (M) receptor agonist methacholine in pithed rats [40].

In conclusion, there is not much evidence that the conduction system of the heart plays a role in the THC-induced tachycardia.

### 4.2. Autonomic System

Several studies shown in Table 2 suggest that the tachycardia induced by THC in humans might result from sympathetic stimulation and/or parasympathetic inhibition, since it was diminished by previous administration of the β-adrenoceptor antagonist propranolol and the M receptor antagonist atropine [107,122,123,124]. However, these results are not consistent. Thus, the THC-induced tachycardia led to a decrease in high-frequency heart-rate variability (HF-HRV), a measure of parasympathetic cardiac control; but to no changes in the pre-ejection period (PEP), a measure of sympathetic cardiac functioning [120]. However, THC shortened PEP in the early study by Kanakis et al. [123]. Moreover, Beaconsfield et al. [108] described an inhibitory effect by propranolol but not by atropine.

The question is whether THC directly acts via the autonomic system and/or via a central mechanism. The results by Gash et al. [110], who found that the maximal increases in HR and plasma noradrenaline (NA) level took place 10 and 30 min after THC smoking, respectively, suggest but do not prove that tachycardia is related to a peripheral mechanism. This conclusion may also be reached from the study by Strougo et al. [117], in which the average population equilibration half-lifes for HR and CNS responses were < 10 min and 40–85 min, respectively.

Provided that the autonomic system is the site involved in THC-induced tachycardia, a direct activation of the sympathetic nervous system and/or an inhibition of the parasympathetic system should occur. To clarify the mechanisms, again in vivo experiments on animals and in vitro experiments on tissues from humans and experimental animals have to be considered.

The possibility that the effect of THC on HR directly involves the peripheral autonomic system was examined in dogs, cats, rabbits and rats. In anaesthetized dogs [149], the THC-induced bradycardia was partially inhibited by spinal section at C2-C4 or by bilateral vagotomy (to destroy the sympathetic and parasympathetic parts of the autonomic nervous system, respectively) and abolished by the combination of both procedures. Vollmer et al. [151] showed that the THC-induced bradycardia in anaesthetized cats was diminished by cervical cardiac denervation but not by vagotomy. In pithed rabbits [139] and in pithed and vagotomized rats [40,160,161,162,163], in which the CNS is mechanically destroyed, neither THC nor one of the synthetic or endogenous cannabinoids under study (i.e., WIN55212-2, CP55940, AEA and MetAEA) produced a fall in HR.

The study by Cavero et al. [149] on dogs also excludes that THC acts via autonomic ganglia. Another two mechanisms have been considered in in vitro studies. Thus, THC might lead to an increased availability of NA due to the inhibition of the neuronal NA transporter or to facilitation of carrier-mediated NA release. The latter two mechanisms are involved in the peripheral effects of cocaine and methamphetamine, respectively, which, as with THC, can elicit a marked tachycardia, sometimes even associated with MI [204,205]. However, an inhibitory effect of THC on the NA transporter in rat hypothalamic synaptosomes occurred at very high concentrations only [206] and THC, CP55940, WIN55212-2, AEA and 2-AG did not facilitate carrier-mediated NA release in rat and mouse renal tissue at all [207]. The results of the latter studies carried out on extracardiac tissues can be transferred to the heart, since the properties of the NA transporter do not differ between tissues.

Although there are some pieces of evidence that THC does not directly act via the autonomic nervous system, more recent data show that there is a direct effect anyway, i.e., via presynaptic receptors. These types of receptors were almost unknown when Cavero et al. [149] and Vollmer et al. [151] carried out their experiments in dogs and cats, respectively. In pithed animal preparations, presynaptic receptors on sympathetic and/or parasympathetic nerve fibers will be overlooked since an impulse flow along the neurones does no longer occur. When, however, the sympathetic outflow of pithed rats [40,160,161] and rabbits [139] was stimulated electrically, a CB_1_ receptor-mediated inhibition of the neurogenic tachycardic response could be demonstrated. In pithed rabbits, a CB_1_ receptor-mediated inhibition of vagal neuroeffector transmission in the heart was also described [139]. In harmony with the latter study, methylatropine, an M-receptor antagonist that does not penetrate the blood–brain barrier, diminished the bradycardia induced by WIN55212-2 in anaesthetized rats [159]. Presynaptic CB_1_-Rs leading to inhibition of atrial NA release have also been identified in vitro in atrial tissue from humans [168], guinea pigs [177] and rats [178]. Presynaptic facilitatory CB_1_-Rs cannot be expected, since CB_1_-Rs are G_i/o_ protein-coupled and only G_s_ and G_q_ protein-coupled receptors lead to facilitation of neurotransmitter release [208]. The other three receptor entities activated by THC, i.e., CB_2_, GPR18 [177] and GPR55, do not serve as presynaptic receptors.

One can expect that sympathetic hyperactivity and reduced parasympathetic transmission are accompanied by several cellular pathologies, typical of MI-induced cardiac injury. Examples are oxidative stress, infiltration of inflammatory cells to the myocardium and peripheral ganglia, elevation of proinflammatory cytokines and nerve growth factor, and activation of satellite glial cells [209].

In conclusion, presynaptic inhibitory CB_1_-Rs are present on the cardiac sympathetic neurones of humans and animals and on the parasympathetic neurones of rabbits; their activation would lead to a decrease and increase in HR. The bradycardia usually elicited by THC in animals might be related to a predominant action on the presynaptic CB_1_-Rs on the sympathetic neurones or by a combined activation of CB_1_-Rs in the brain and in the autonomic system. The fact that tachycardia occurs in humans instead may be related to a stimulatory input from the CNS which overrides the brake due to the inhibitory CB_1_ receptors on the sympathetic nerve endings. The potential role of presynaptic inhibitory CB_1_-Rs on the cardiac human parasympathetic neurons has so far not been examined.

### 4.3. Central Nervous System

In order to obtain a deeper insight into the brain mechanisms involved in the effect of THC on HR, experiments are of interest in which THC or another cannabinoid was administered to the cerebrospinal fluid (CSF) or directly into brain sites involved in cardiovascular regulation. In studies on dogs [149], cats [151], rabbits [140,141] and rats [210,211,212] in which THC or another cannabinoid agonist was injected into the cerebral circulation or into the CSF, a bradycardia occurred consistently (Table 5). Only in one study on anaesthetized rats, an agonist with preference for CB_1_ receptors did not affect HR at all [210].

**Table 5:** Cardiovascular effects of acute cannabinoid administration into the cerebral circulation, the cerebrospinal fluid or directly into selected brain areas.

Species	Conscious/ Anaesthetized with	Site of Injection	Drug under Study	Dose (nmol/rat), if Not Stated Otherwise	Effects	Possible/Suggested Mechanisms and Involvement of CB_1_-Rs/CB_2_-Rs/Others if Determined ^1^	References
rabbits	conscious	intracisternal	CP or WIN-2 CP or WIN-2 WIN-3	0.1 and 1 µg/kg 10 µg/kg 0.1, 1 or 10 µg/kg	↓HR, ↑RSNA, ↑plasma NA ↓HR, ↑RSNA, ↑plasma NA + ↑BP no effects	↓HR and ↑BP, related to central CB_1_-Rs (diminished by i.v. RIM, not shared by inactive WIN-3) ↓HR related to muscarine receptors (also reduced by atropine)	[140,141]
mongrel dogs	pentobarbital	head circulation	THC	2.5 mg/kg	↓HR	THC-induced bradycardia has a central origin and involves an alteration of the central autonomic outflow regulating HR	[149]
cats	chloralose	lateral cerebral ventricle	THC	2 mg/kg	↓HR; ↔ BP	THC-induced bradycardia mediated centrally and not associated with a substantial reduction in BP	[151]
WKY	urethane	i.c.v.	ACEA	1400	↔ HR, ↔ BP, ↔plasma NA and Adr		[210]
Wistar rats	urethane	intracisternal	WIN-2 WIN-3	both 1, 3, 10 and 30 µg/kg	WIN-2 unlike WIN-3: ↓HR, ↑BP and ↑plasma NA	CB_1_-Rs in the brain stem enhance cardiac vagal tone and sympathetic tone (all effects diminished by RIM)	[211]
Sprague Dawley rats	conscious	intracisternal	WIN-2	23 and 70	immediate ↓HR but delayed ↑BP and ↑plasma NA (maximum at 10 min)	↓HR, ↑BP and ↑plasma NA depend on CB_1_-Rs (reduced by AM251); ↑BP and ↑plasma NA but not ↓HR reduced by GABA_A_-R agonist muscimol	[212]
Sprague Dawley rats	conscious	**RVLM**	WIN-2	0.1, 0.2, 0.3	↑HR, ↑BP	difference in the HR response between Ibrahim and Abdel-Rahman [204] vs. [205] might be caused by the localized effect of WIN-2 within the RVLM compared to a more widespread effect after intracisternal administration	[213]
Wistar rats	conscious	RVLM	ACEA AM251	0.00005 0.00025	↑HR, ↑BP, ↑RSNA ↓HR, ↓BP, ↓RSNA	↑HR and ↑BP mediated by CB_1_-Rs (reduced by AM251); CB_1_-Rs tonically active (AM had effects opposite in direction to those of ACEA)	[94]
Wistar rats	conscious	RVLM	AM251	0.00025	↓HR and ↓BP	CB_1_-Rs activated tonically by eCBs (cf. study by Wang et al. [94])	[214]
Sprague Dawley rats	conscious	RVLM	abn-CBD NAGly O-1918	0.65, 1.3, 2.5 1.4, 2.8, 5.5, 11 0.7, 1.4, 2.8	↑HR, ↓BP ↔ HR, small ↓BP ↓HR, ↑BP	GPR18-Rs might mediate tachycardia and hypotension; probably activated by eCBs (O-1918 had effects opposite in direction to those of abn-CBD)	[215]
Sprague Dawley rats	urethane	RVLM	WIN-2 HU-210	0.00005, 0.0005 or 0.005 0.0005	both agonists: ↔ HR, ↑ BP and ↑ sSNA	central sympathoexcitation mediated by CB_1_-Rs (reduced by AM281)	[216]
Wistar rats	urethane	RVLM	WIN-2	12	↔HR; slight ↓BP; ↔plasma NA	not examined	[159]
Sprague Dawley rats	pentobarbital	**dPAG**	AEA	0.0018	↑HR, ↑BP, ↑RSNA; higher baseline HR connected with increased AEA content and decreased FAAH activity	eCBs can lead to sympathoexcitation via modulation of GABAergic inhibition by CB_1_-Rs at the level of the dPAG (responses reduced by AM281 and the GABA_A_-R antagonist gabazine)	[217,218,219]
Wistar rats	urethane	**PVN**	MethAEA CP *MethAEA* +AM251 *or* *CP* +AM251 *or* +AM6545	10 or 0.1 *doses of agonists* *as above;* antagonist doses (mg/kg): AM251 1.7 i.v. AM6545 8.3 i.p.	↓HR, ↓BP ↑HR, ↑BP	the centrally induced ↑HR and ↑BP is mediated by CB_1_-Rs in the PVN (reduced by AM251 given into the PVN) and can be masked by peripheral CB_1_-Rs; the direction of the response (↑ or ↓ of HR and BP) probably depends on the sympathetic tone	[220,221]
Wistar rats	urethane	PVN	CP + AM251 1.7 mg/kg i.v.	10	↑HR, ↑BP	pressor response of CP (after blockade of peripheral CB_1_-Rs by AM251) mediated via NMDA-, GABA_A_-, β_2_-, TP-, AT_1_-Rs and NO (antagonized by the respective inhibitors given i.v.)	[220,221]
Wistar rats	conscious	**BNST**	AM251 URB597	0.001, 0.03, 0.1 0.03	↑HR but not ↑BP induced by restraint stress increased by AM251 and decreased by URB597	CB_1_-Rs and eCBs play a role in cardiac responses during stress via modulation of NMDA receptors in BNST and GABA_A_-Rs in the lateral hypothalamus; amplificatory effect of AM251 reduced by the respective antagonists LY235959 and SR95531	[222,223,224]
dog	α-chloralose + urethane	**NTS**	WIN-2 RIM	1.25–1.50 pmol 2.5–3.0 pmol	↔BP, ↔BRS ↔BP, ↔BRS		[225]
Sprague Dawley rats	urethane	NTS	WIN-2 CP AM281	10 10 14	↔ HR, ↓BP ↔ HR, ↓BP, ↔BRS ↔ HR, ↔ BP, ↔BRS	CB_1_-Rs in NTS do not modulate HR and baroreflex sensitivity	[226]
Sprague Dawley rats	pentobarbital	NTS	AEA AM404 *(AEA transport* *inhibitor)*	0.0025 0.0035	both drugs: ↔HR; ↔BP ↑BRS	CB_1_-Rs activated by eCBs in the NTS may have presynaptically attenuated GABA release, leading to enhanced BRS (effects of AEA inhibited by RIM and GABA_A_-R antagonist bicuculline)	[41,227]
Wistar rats	conscious	**vMPFC**	AM251	0.1	↔ HR and ↔ BP by itself; ↑BRS	CB_1_-Rs reduce the cardiac responses of the baroreflex	[228]

^1^ CB-R antagonists were mentioned only if their cardiac effects were determined independent of CB-R agonists. Antagonists did not modify cardiac parameters by themselves, unless stated otherwise. ↑increase; ↓decrease; ↔no effect; β_2_-R, β_2_-adrenergic receptor; abn-CBD, abnormal cannabidiol; ACEA, arachidonyl-2-chloroethylamide; Adr, adrenaline; AEA, anandamide; AT_1_-R, angiotensin II receptor type 1; BNST, bed nucleus of stria terminalis; BP, blood pressure; BRS, baroreceptor-reflex sensitivity expressed as the ratio of the HR change over the change in the mean BP; CB_1_-R_,_ cannabinoid CB_1_ receptor; CB_2_-R_,_ cannabinoid CB_2_ receptor; CP, CP55940; dPAG, dorsal periaqueductal gray; eCBs, endocannabinoids; FAAH, fatty-acid amide hydrolase; GABA, γ-aminobutyric acid; GABA_A_-R, γ-aminobutyric acid type A receptor; GPR18, G protein-coupled receptor 18; HR, heart rate; i.c.v., intracerebroventricular; i.p., intraperitoneal; i.v., intravenous; MethAEA, methanandamide; NA, noradrenaline; NAGly, N-arachidonoyl glycine; NMDA-R, N-methyl-D-aspartate receptor; NTS, nucleus tractus solitarii; PVN, paraventricular nucleus of hypothalamus; R, receptor; RIM, rimonabant; RSNA, renal sympathetic nerve activity; RVLM, *rostral ventrolateral medulla;* sSNA, splanchnic nerve activity; THC, Δ^9^-tetrahydrocannabinol; TP, thromboxane receptor; URB597, inhibitor of fatty-acid amide hydrolase; vMPFC, ventromedial prefrontal cortex, WIN-2, WIN55212-2; WIN-3, WIN55212-3; WKY, Wistar-Kyoto rats.

An entirely different picture emerged when THC or an agonist was injected into brain areas relevant for cardiovascular regulation; all studies of that kind were performed on rats (Table 5; experiments involving the nucleus tractus solitarii (NTS) and the ventral medial prefrontal cortex (vMPFC) will be discussed under Section 4.4). Microinjection of the cannabinoid into the rostral ventrolateral medulla (RVLM) caused tachycardia [94,213], bradycardia [214] or did not affect HR at all [159,216]. In one study, injection of a GPR18 agonist into the RVLM increased HR [215]. HR was increased following microinjection of AEA into the dorsal periaqueductal grey (dPAG; [217,218,219]) but decreased upon microinjection of an inhibitor of AEA degradation into the bed nucleus of the stria terminalis (BNST; [222,223,224]); both effects were mediated via CB_1_-Rs.

In our own studies on the paraventricular nucleus of the hypothalamus (PVN; [220,221]; Table 5), cannabinoids led to brady- or tachycardia, dependent on the experimental conditions. Microinjection of the cannabinoid CP55940 into this brain region led to bradycardia, which was antagonized by the CB_1_-R antagonist AM251 given into the PVN. When AM6545, a CB_1_-R antagonist which does not penetrate the blood–brain barrier, was injected i.v., CP55940 microinjected into the PVN elicited tachycardia instead. One interpretation of these findings might be that CP55940 increases the sympathetic outflow, which is, however, inhibited by cardiac presynaptic inhibitory CB_1_-Rs, eventually leading to bradycardia. If the “brake” is removed by blockade of these receptors, tachycardia will occur instead. The latter studies are somewhat reminiscent of the paper by Szabo et al. [139] on conscious rabbits, in which WIN55212-2 injected i.v. to conscious rabbits produced bradycardia at lower doses (0.005 and 0.05 mg/kg) but tachycardia at the highest dose (0.5 mg/kg). One might assume that the bradycardia is the result of the central activation of the parasympathetic outflow but is reversed into a tachycardia when the presynaptic CB_1_-Rs on the vagal nerves are strongly activated.

THC does not only act via the autonomic nervous system but also activates the hypothalamus/pituitary/adrenal cortex (HPA) axis, eventually leading to an increased cortisol secretion [229]. Cortisol in turn sensitizes the adrenoceptors which lead to tachycardia and BP increase. THC acts via different pathways in the brain, namely via noradrenergic neurones projecting from the locus coeruleus and serotoninergic neurones projecting from the raphe nuclei to the PVN. In addition, the PVN, which represents the origin of the HPA axis, is also activated by neurones originating in higher brain regions (reviewed in El Dahan et al. [230]).

In conclusion, there is good evidence that the effect of THC on HR is the result of a combined action between central CB_1_ receptors in areas of the brain involved in cardiovascular regulation and peripheral presynaptic inhibitory CB_1_ receptors on sympathetic and/or parasympathetic nerve fibers. It is interesting that even within the same animal species cannabinoids can elicit either brady- or tachycardia. In humans, the interplay between central and peripheral CB_1_ receptors appears to be such that tachycardia will be the only effect. Tachycardia is further increased by a THC-driven rise in cortisol secretion.

### 4.4. Baroreceptor Reflex

Administration of conventional (rapid release) formulations of the Ca^2+^ channel blocker nifedipine led to marked tachycardia sometimes associated with MI [231]; this side effect is related to unloading of the baroreceptor reflex due to an abrupt fall in blood pressure. Table 2 shows that cannabinoids, although they decreased blood pressure in two studies on conscious humans, usually increase blood pressure or leave it unaffected. Moreover, the dose-dependent increases in BP induced by THC are better correlated to changes in HR than to the dose [104].

There are few studies on anaesthetized animals in which the effect of topical administration of cannabinoids into the NTS on HR and on baroreceptor sensitivity was examined (Table 5). HR was not affected in rats [41,226,227]. Baroreceptor sensitivity was not affected in dogs [225] and in rats in the study by Durakoglugil and Orer [226], increased in the studies by Seagard et al. [227] and Brozoski et al. [41] and even decreased in the study by Lagatta et al. [228]. In the latter study, which was performed on conscious rats and in which the CB_1_-R antagonist AM251 was topically administered to the vMPFC, HR remained constant but baroreceptor sensitivity increased, suggesting that the CB_1_-Rs in this brain region decrease its sensitivity [228].

In conclusion, it is unlikely that the baroreceptor reflex plays a role in the tachycardia elicited by THC and other cannabinoids.

## 5. Thrombus Formation and Coronary Constriction?

Since THC can lead to MI associated with tachycardia, the question arises whether THC also influences the two major causes of MI, i.e., thrombus formation and coronary constriction.

Multiple case reports have linked marijuana to thrombus formation, leading to acute MI [13], and the authors quote the publication by Deusch et al. [91] on human platelets in vitro in which CB_1_- and CB_2_-Rs are expressed and THC enhances expression of the platelet fibrinogen receptor (glycoprotein IIb-IIIa) and P-selectin; experiments with CB-R antagonists were not performed in that study. In other studies, THC by itself failed to modify the aggregation of human platelets [186] and even decreased aggregation of human and rabbit platelets induced by various factors [182]. Agonists such as ACEA, HU-210 and JWH-015 also failed to modify the aggregation of human [187,188] or rabbit platelets [190]; the same held true for the CB_1_- and CB_2_-R antagonists AM251 and AM630 [186,189] and for LPI (endogenous ligand of the GPR55), which, in addition, decreased the ADP-induced platelet aggregation [26]. Only the endocannabinoids 2-AG, AEA and virodhamine clearly activated human platelets and stimulated their aggregation [185,186,187,188]; however, in a manner dependent on thromboxane A_2_, which cannot be formed from THC and synthetic cannabinoids (for details, see Table 4). In the only in vivo study, WIN55212-2 given i.p. failed to affect thrombus formation in the ear venules of hairless mice whereas AEA reduced it, again by an arachidonic acid-derived agent ([164]; Table 3).

The possibility that THC leads to alterations of the vascular wall and eventually to atherogenesis and atherotic plaque rupture was considered in human cells. CB_1_-R activation increases the formation of reactive oxygen species (ROS) and accumulation of lipid droplets in macrophages, ROS production and injury of endothelial cells and ROS formation and migration of vascular smooth-muscle cells. By contrast, CB_2_-Rs usually influence the three cell types in an opposite direction, i.e., they counteract the formation of lipid droplets in macrophages, inhibit the adhesive and infiltrative properties of endothelial cells and inhibit migration of vascular smooth-muscle cells (reviewed in El Dahan et al. [230]).

In conclusion, taking into account the experimental studies discussed above, there is not much evidence to suggest that THC and synthetic cannabinoids (unlike endocannabinoids) can lead to platelet aggregation as a process eventually leading to thrombus formation, but they may promote atherogenesis and atherotic plaque rupture as a prerequisite of thrombus formation. Whether a detrimental effect on the wall of the coronary arteries will really occur in vivo is, however, unclear since CB_2_-R activation, unlike CB_1_-R, has effects in the opposite direction.

Coronary flow (CF) is impaired by tachycardia, since blood supply to the coronary arteries can occur only during the diastole of the heart action. Consequently, in the rat perfused spontaneously beating heart, THC caused tachycardia that was accompanied by a decrease in coronary flow [169]. In this section, we will consider the possibility that THC and related compounds have a direct effect on the coronary arteries.

THC reduced CF in the rat perfused heart in which vasopressin (VP) was given to induce coronary tone [175]. By contrast, other cannabinoids enhanced CF both under standard conditions [77,174,176] and under VP ([175]; for details, see Table 4). The increase in CF was mainly connected to changes in ventricular performance measured as a decrease in left ventricular developed pressure (LVDP, which is obtained by subtracting the end-diastolic pressure from the left ventricular systolic pressure; LVSP); this phenomenon might explain the increase in CF [77,174]. However, the increase in CF in coronary arteries preconstricted with VP was associated with an enhanced LVSP [175].

Which receptors are involved in the above effects? Surprisingly, the decrease in CF by THC was not antagonized by CB_1_- and CB_2_-R antagonists. On the other hand, CB_1_-Rs are involved in the enhancement of coronary vasodilation, since CB_1_-R antagonists such as rimonabant, AM251 and O-2050 but not the CB_2_-R antagonist SR144528 or the GPR18 antagonist O-1918 showed an antagonistic effect [77,175]. For the ACEA-induced increase in CF, Ford et al. [174] suggest the involvement of a novel site since this effect was reduced by rimonabant, AM281 (CB_1_-R antagonists) and SR144528 (CB_2_-R antagonist), but not by AM251 (CB_1_-R antagonist) and AM630 (CB_2_-R antagonist).

The results of experiments on isolated rat coronary arteries [90] are consistent with those obtained on isolated hearts. Thus, WIN55212-2 elicited vasodilation and this effect was antagonized by two CB_1_-R antagonists [90].

In conclusion, a direct dilatory effect of cannabinoids on coronary arteries has been shown in one in vitro model of the rat only. Although most cannabinoids increase CF, THC itself showed an inhibitory effect.

## 6. Increased Energy Demand and Decreased Energy Supply?

Tachycardia, thrombus formation and/or coronary constriction have been discussed as factors involved in the development of acute MI accompanying the use of THC or related compounds. The question arises whether other factors may contribute. An increase in energy demand and/or a decrease in energy supply may play a role.

An increase in energy demand might be caused by a positive inotropic effect of cannabinoids. Indeed, in isolated rat left atria the CB_2_-R agonist JWH-015 and AEA, examined in the presence of a CB_1_-R antagonist, induced a positive inotropic effect which is mediated by CB_2_-Rs [180]. Moreover, Walsh et al. [87] concluded from their experiments on GPR55-deficient mice that GPR55 increases the adrenoceptor-mediated positive inotropic response.

By contrast, a negative inotropic effect of THC, ∆^8^-THC and HU-210 was obtained in the perfused rat heart (Table 4; [157,170,173]); in those studies, however, the authors did not use CB-R antagonists to determine the type of receptors involved. In the study by Sterin-Borda et al. [180] in rat left atria, the CB_1_-R agonist ACEA alone, as well as AEA in the presence of a CB_2_-R antagonist, had a negative inotropic effect. Likewise, AEA had a negative inotropic effect in human right atrial muscle, which was mimicked by its stable analogue MethAEA and by HU-210 and antagonized by the CB_1_-R antagonist AM251 [70]. The latter increased contractility of human right atrial muscles by itself [70], suggesting that these receptors are activated by endocannabinoids or are constitutively active. The lack of a positive inotropic effect of AEA in isolated human, as opposed to rat, cardiac tissue [70] may be due to species differences. However, Bonz et al. [70] have not examined the effect of AEA in the presence of a CB_1_-R antagonist or of a selective CB_2_-R agonist. An opposite influence of CB_1_-R and CB_2_-R activation in the rat heart might be the reason for the lack of changes in contractile function of the isolated rat heart in response to AEA alone [180] and in rat ventricular myocytes in response to the CB_1_-/CB_2_-R dual agonist CB13 [18]. Another two studies are difficult to interpret. Thus, in the rat perfused heart, both a CB_1_- and a CB_2_-R antagonist showed a negative inotropic effect [172] and in rabbit left ventricular myocytes the CB_2_-R agonist A-955840 had a CB_1_-R- and CB_2_-R-independent negative inotropic effect [38].

In conclusion, there is no evidence that THC and other cannabinoids elicit a positive inotropic effect in the human heart.

A decrease in energy supply might be caused by an impairment of oxygen transport by cannabis use. Since tobacco smoking is associated with an increased carboxyhemoglobin level resulting in decreased cardiac oxygen supply (reviewed in Dorey et al. [232]), the frequent combination of tobacco and cannabis smoking can explain the impairment of oxygen supply in many instances. However, cannabis use per se can also lead to significantly increased expired carbon oxide concentrations, provided that THC was administered by smoking but not when it was vaporized or taken orally ([129]; Table 2). An increase in serum carboxyhemoglobin level was also observed in an animal study, i.e., in mice “smoking” cannabis cigarettes via a special smoke-inhalation system ([148]; Table 3).

Smoking cannabis can decrease the oxygen transport to the heart and, in this respect, changes in mitochondrial oxygen consumption in response to cannabinoids are of interest. However, the results obtained so far are contradictory. Thus, on the one hand, THC or AEA and HU-210 not only at high (100–120 or 1–20 μM) but even at low (0.1 or 0.2 μM) concentrations led to a decrease in oxygen consumption in bovine [193], rat [194] and mouse [191,192] cardiac tissue or mitochondria. The latter was connected with a lower mitochondrial membrane potential and an enhanced mitochondrial hydrogen peroxide production (Table 4). The detailed mechanism(s) of the above changes have so far not been examined. Although CB_1_Rs were detected in cardiac mitochondria, Mendizabal-Zubiaga et al. [192] excluded CB_1_-Rs, since similar changes were observed in CB_1_^–/–^ and CB_1_^+/+^ mice. By contrast, a detailed analysis of the toxic effects of a wide range of THC concentrations (1–500 μM) on isolated rat-heart mitochondria failed to detect any changes regarding an enhanced production of reactive oxygen species or lipid-peroxidation products, mitochondrial swelling or changes in mitochondrial membrane potential [195]; the authors even concluded that THC may be helpful in reducing mitochondrial toxicity. Moreover, the dual CB_1_/CB_2_ receptor agonist CB13 prevented cardiac mitochondrial dysfunction (such as membrane depolarization and decreased mitochondrial bioenergetics) induced by endothelin-1 in neonatal rat ventricular myocytes [196].

Even if a direct effect of marijuana or cannabimimetics on mitochondrial function is controversial, indirect effects should be considered as well. Thus, all potential mechanisms involved in cardiac injury such as tachycardia, constriction of coronary artery and platelet aggregation, changes in action potential (mainly disturbances in calcium homeostasis), i.e., pathological conditions characterized by the deprivation of oxygen supply to cardiomyocytes, may impact adversely on mitochondrial function [233]. There is increasing evidence for an important role of cardiac (e.g., [234,235,236]) and coronary microvascular [237,238] mitochondria in MI.

In conclusion, independent from tobacco smoking, cannabis smoking can lead to an increase in carboxyhemoglobin and a subsequent reduction in oxygen transport. Although controversial data exist as to whether THC affects mitochondrial respiration directly, an indirect detrimental effect (e.g., due to tachycardia) is very likely.

## 7. Other Factors

MI can lead to life-threatening arrhythmias, and for this reason possible effects of THC and other cannabinoids on the conduction system of the heart are of great relevance.

In the study by Miller et al. ([125], Table 2), i.v. THC enhanced sinus automaticity and facilitated sinoatrial and atrioventricular nodal conduction in humans in vivo, most probably representing the positive chronotropic and positive dromotropic effect elicited by noradrenaline as a result of sympathetic stimulation; intra-atrial and intraventricular conduction was not affected. It is very plausible that noradrenaline will stimulate the cardiac conductive system, thereby eventually leading to a proarrhythmogenic effect under pathological conditions.

On the other hand, some effects on cardiac ion channels in cardiomyocytes may be beneficial in tachyarrhythmias. As shown in Table 4, cannabinoids might exert an antiarrhythmic effect related to the inhibition of cardiac voltage-gated inward L-type Ca^2+^ currents; this verapamil-like effect was shown in rat tissue and is related to the activation of CB_1_-Rs [199]. The suppression of cardiac Na^+^/Ca^2+^ exchanger (NCX1)-mediated currents in rat ventricular cardiomyocytes may contribute to an antiarrhythmogenic effect of JWH-133 under ischemic conditions (mediated via CB_2_-Rs; [201]). Moreover, prolongation of the AP in response to THC was found in sheep Purkinje fibers (receptor not determined; [197]). The synthetic agonist CB13 inhibited the tachypacing-induced shortening of the rat atrial effective refractory period protecting against atrial fibrillation (receptor not identified; [80]).

By contrast, a decrease in AP duration was observed in rabbit sinoatrial-node samples in response to AEA (CB_1_-Rs involved; [198]) and in rabbit Purkinje fibers in response to a high concentration of JWH-030 (mechanism unclear; [156]). Moreover, the endogenous agonist of GPR55 receptors, l-α-lysophosphatidylinositol (LPI), increased Ca^2+^ entry via L-type Ca^2+^ channels in rat cardiomyocytes [86]; the involvement of GPR55 receptors has been proven using an appropriate antagonist (Table 4).

In conclusion, although THC may lead to tachyarrhythmia as a result of a high NA level, evidence for direct anti- and proarrhythmogenic effects is restricted to in vitro studies on preparations from animals.

There may be other (adverse) cardiac effects of THC or synthetic cannabinoids suggested by in vitro experiments (Table 4): (1) THC, at a high concentration of 30 μM, acted cardiotoxically and stopped cardiac activity in the perfused rat heart [169]; (2) an enhanced apoptosis caused by endoplasmic reticulum stress in mouse HL-1 atrial cardiomyocytes occurred after treatment with high THC concentrations of 10 and 30 μM [203]; (3) cell viability in H9c2 cells (rat cardiomyoblast cell line) decreased in response to the synthetic cannabinoids JWH-030, JWH-210, JWH-250 and RCS-4 [156] and (4) the primary metabolites of THC i.e., 11-hydroxy-Δ^9^-THC (THC-OH) and 11-nor-9-carboxy-Δ⁹-tetrahydrocannabinol (THC-COOH), but not the parent compound THC itself [202].

The mechanism(s) of cardiotoxicity are still not entirely clear. When the study by Nahas and Trouve [169] appeared, CB-Rs had not yet been deciphered. The use of selective CB_1_- and CB_2_-R antagonists revealed that the apoptotic effect of THC is neither CB_1_- nor CB_2_-R-related [203] whereas CB_2_-Rs are involved in the effect of JWH-030 on cell viability [156]. Although THC is devoid of an inhibitory effect on cell viability in H9c2 cells, it may have such an effect in vivo. One can expect that, unlike in a cell line, THC is metabolized to THC-OH and THC-COOH. The metabolites, however, do not possess any affinity for CB_1_- or CB_2_-Rs.

In conclusion, some interesting cardiotoxic effects of THC and synthetic cannabinoids have been shown in vitro but it is unclear to which extent they play a role in humans in vivo.

## 8. General Conclusions

**Figure 2:**
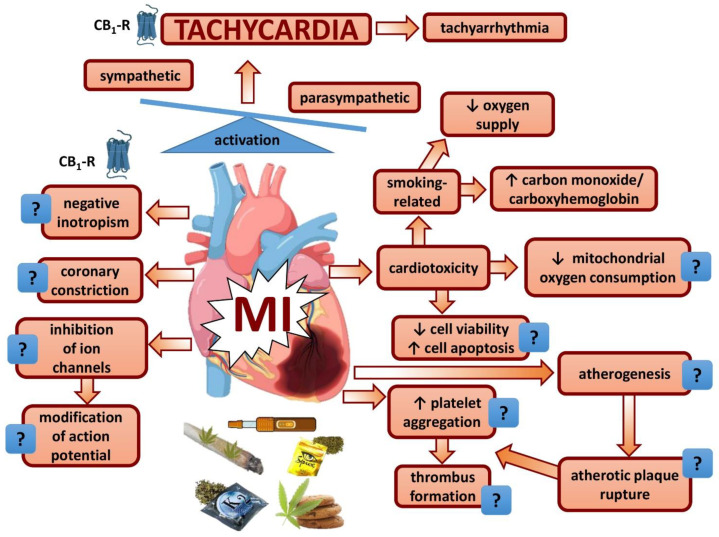
Effects of cannabinoids on the heart possibly implicated in myocardial infarction. ?, pathophysiological relevance plausible but not supported by appropriate studies; CB_1_-R, cannabinoid CB_1_ receptor; MI, myocardial infarction.

Cannabis contains Δ^9^-tetrahydrocannabinol as its major psychotropic principle and, with respect to vegetative effects, its use for recreational purposes has been considered safe over a long time period. In recent years, however, an increasing number of studies revealed serious cardiovascular effects, even including acute myocardial infarction (MI) in healthy young people; indeed, cannabis has been listed among the risk factors of MI. The potential mechanisms induced by exposure to THC and other cannabimimetics triggering MI are shown in Figure 2. MI related to cannabis use is associated with tachycardia. Tachycardia is the most reliable biomarker of cannabis use and occurs independent of the route of administration. The reason why cannabis elicits tachycardia in humans but almost exclusively bradycardia in animals is unclear but may have to do with the relatively low heart rate level in humans. One explanation for the difference between humans and animals might be that the cannabinoid CB_1_ receptor-driven central stimulation of the sympathetic system is inhibited markedly by presynaptic inhibitory CB_1_ receptors on the sympathetic nerve fibers in animals but only slightly in humans. Cannabis use is frequently associated with tobacco smoking, thereby increasing the risk to develop MI. However, it is questionable whether the two most typical pathogenetic factors for the development of MI, i.e., thrombus formation and coronary constriction, play a role in the case of cannabis-related MI, at least not on the basis of the few studies available. Administration of cannabis by smoking but not by other routes impairs energy supply by increasing the formation of carboxyhemoglobin; impairment of mitochondrial respiration is an additional factor. Worsening of MI by an increased energy demand because of a positive inotropic effect is unlikely. Proarrhythmogenic effects of cannabis per se are unlikely but may appear as a consequence of increased noradrenaline levels associated with tachycardia. The increasing use of cannabis preparations for recreational but also for therapeutic purposes warrants each effort to further elucidate cardiovascular mechanisms, in order to avoid severe side effects.

## Figures and Tables

**Table 1:** Reviews from the past 5 years highlighting the potential impact of cannabis intake on the increase in risk of myocardial infarction and other severe cardiovascular disorders.

*Type of study* Number of Patients/ Sample/Evaluation Period	Results	Final Conclusion	References
*Systematic review* 115 articles (81 case reports, 29 observational studies, 3 clinical trials and 2 experimental studies). 116 individuals in case reports, mean age 31 (2011–2016)	- individuals (82% men) mainly suffered from ischemic strokes (27 cases) or myocardial infarctions (33 cases) - 11 deaths from cardiovascular disease following exposure to cannabis-based products	association between exposure to cannabis-based products and cardiovascular disease; evidence stronger for ischemic strokes than for any other cardiovascular diseases	[54]
*Epidemiologic study from NIS* 2,451,933 acute MI patients, mean age 49 (2010-2014)	cannabis use raised the risk of acute MI by 3–8%	importance of patient history including recreational drug use in identifying the etiology of an unexplained MI	[55]
*Epidemiologic study from NIS* 316,397 patients, aged 18–55 (2009–2010)	heart failure (1.4 vs. 1.2%), cerebrovascular accidents (1.03 vs. 0.62%), CAD (5.0 vs. 4.6%) and sudden cardiac death (0.21 vs. 0.17%) significantly higher in cannabis users	cannabis use is an independent predictor of heart failure and cerebrovascular accidents	[56]
*Retrospective cohort analysis* 292,770 patients with a history of cannabis abuse and 10,542,348 age- and sex-matched controls, mean age 37 (2011–2016)	3-year cumulative incidence of MI significantly higher in the cannabis-abuse group than in controls (1.37% vs. 0.54%); most pronounced risk in the young and middle-aged	cannabis abuse may be associated with an elevated risk of MI independent of other cardiovascular risk factors, with higher relative risk in women and younger age groups	[53]
*Epidemiologic study from NIS* 52,290,927 hospitalized patients, aged 18–39 years (2007–2014)	hospitalizations among young cannabis users, compared to non-users increased by 50% for MI, 79% for arrhythmias, 300% for stroke and 75% for venous thromboembolic events	young cannabis users are more at risk in hospitalizations for acute MI, arrhythmia, and stroke	[57]
*Scoping review* 92 articles (1 randomized control trial, 4 systematic reviews, 19 literature reviews, 11 large database reviews, and 42 case reports/series) (2003–2018)	- 51 cases of cannabis-related MI - 71% of patients suffered from ST elevation MI (STEMI) - 22% of patients presented with an out-of-hospital cardiac arrest or had an arrest prior to intervention; half of these patients died	- cannabis-induced MI especially prevalent among young healthy patients, presenting shortly after use - users aged 18–36 years had an increased risk for acute coronary syndrome compared to non-users	[13]
*Epidemiologic study from NHANES* 89.6 million adults who reported marijuana use (2005–2016)	2015–2016: 2 million (2.3%) cannabis users have a cardiovascular disease	screening for marijuana use should be conducted in young patients with cardiovascular disease	[14]
*Epidemiologic study from NIS* 3,307,310 hospitalizations were noted with cannabis use disorder, mean age 36 (2007–2014)	among cannabis users 0.7% (n = 24,148) had malignant hypertension	it is necessary to develop an intervention to raise awareness regarding the deleterious effect of cannabis use and to curtail additional healthcare costs	[58]
*Retrospective longitudinal cohort study* 18,653 adult cannabis patients were matched to 51,243 controls, aged 31–60 years (2014–2017)	incidence rates for ACS or stroke were 7.19/1000 and 5.67/1000 person/year in the cannabis and control group	medical cannabis authorization associated with increased risk of ED visits or hospitalization for cardiovascular events including stroke and ACS	[59]
*Systematic review* 16 studies: 4 cohort, 8 case-control studies, 1 case-crossover study, 2 randomized controlled trials and 1 descriptive study with 10 to 118,659,619 participants (1970–2018)	marijuana use was related to an increased risk of MI in 2 studies and of stroke in 6 studies (within 24 h)	marijuana users may be at increased risk of cardiovascular events	[60]
*Epidemiologic study from NHANES* 3634 participants (1988–1994)	about 26.0% (*n* = 900) of participants (mean age 48) were ever cannabis users and 15.5% (*n* = 538) had myocardial injury	cannabis use increased risk of myocardial injury among people without cardiovascular disease; effect increased by coexistent hypertension	[61]
*Epidemiologic study from BRFSS* 33,173 young adult par-ticipants, 4610 (17.5%) respondents reported recent cannabis use (2017–2018)	MI more frequent among recent cannabis users (61 of 4610, 1.3%) relative to non-users (240 of 28,563, 0.8%) history of MI associated with cannabis use of more than 4 times per month and with smoking as the primary method of consumption	- association between recent cannabis use and history of MI in young adults - increasing cannabis use in an at-risk population might have negative implications for cardiovascular health	[62]

ACS, acute coronary syndrome; BRFSS Behavioral Risk Factor Surveillance System Survey; CAD, coronary artery disease; ED, emergency department; MI, myocardial infarction; NHANES, National Health and Nutrition Examination Survey; NIS, National Inpatient Sample; STEMI, ST segment elevation myocardial infarction.

**Table 3:** Cardiac effects of acute cannabinoid administration in experimental animals in vivo.

Species	Anesthesia	Agonist	Dose (mg/kg) and Route of Administration ^1^	Effects	Mechanisms and Involvement of CB_1_-Rs/CB_2_-Rs/Others if Determined	References
rhesus monkeys	conscious	THC	0.5 i.v.	↑HR	antagonists not used	[133]
rhesus monkeys	conscious	THC	0.75–4 i.p.	↓HR	antagonists not used	[134]
rhesus monkeys	conscious	THC WIN-2	0.1–10 i.m. 0.1–10 i.m.	↓HR	bradycardia is mediated viaCB_1_-Rs (prevented by RIM)	[135]
rhesus monkeys	conscious	RIM	3.2 i.v.	↑HR	CB_1_-Rs responsible for bradycardia are activated via endocannabinoids	[136]
mongrel dogs	conscious	THC	1 and 2.5 i.v.	↓HR and CO dose-dependent; ↑RVSW	antagonists not used	[137]
beagle dogs	conscious	Sativex^®^ (CBD: THC)	spray; max. plasma levels 10.5:18.5 ng/mL	↔HR	antagonists not used	[138]
rabbits	conscious	WIN-2	0.005 and 0.05 i.v. 0.5 i.v.	↓HR ↑HR	both bradycardia and tachycardia are mediated by CB_1_-Rs, since RIM reduced bradycardia and reversed tachycardia	[139]
rabbits	conscious	CP WIN-2 WIN-3	each 0.0001, 0.001, 0.01 i.c.	↓HR; dose-dependent ↓HR; dose-dependent ↔HR	bradycardia related to an increase in cardiac vagal activity and CB_1_-Rs (prevented/diminished by i.v. atropine and RIM)	[140,141]
Wistar rats	conscious	THC	4, 6, 8 i.p.	↓HR	antagonists not used	[142]
Sprague Dawley rats	conscious	WIN-2	0.005, 0.05 0.25 i.v.	↔HR ↓HR	since WIN-2 increased BP authors suggested that the clear dissociation between the effects of WIN-2 on BP and HR are consistent with the bradycardia being mediated centrally	[143]
Sprague Dawley rats	conscious	THC JWH-018 AM2201 XLR-11 CP	0.3–3.0 s.c. 0.18–0.56 0.1–0.3 0.1–3.0 0.01–0.1	↔HR ↔HR ↔HR ↔HR ↔HR	THC, CP and the synthetic cannabinoids JWH-018, AM2201 and XLR-11 increase BP only	[144]
Wistar rats	conscious	O-1602	0.25 i.a.	↔HR	antagonists not used	[145]
CB_1_^+/+^ and CB_1_^−/−^ mice	conscious	AEA WIN-2	0.25 2 i.v.	↓HR but only in CB_1_^+/+^ but not in CB_1_^−/−^	bradycardia is mediated via CB_1_-Rs	[146]
CB_1_^+/+^ and CB_1_^−/−^ mice	conscious	deletion of CB_1_-R	-	basal HR was higher in CB_1_^−/−^ than in CB_1_^+/+^ but only during active period	eCBs induce bradycardia via the activation of CB_1_-Rs but only during active period	[147]
mice GPR55^+/+^ GPR55^−/−^	conscious or isoflurane	deletion of GPR55	-	in GPR55^−/−^: ↓basal HR ↑LVEDV, LV mass, heart weight, ↔LVID, CO, EF	GPR55 affects preload and chronotropy	[85]
mice	conscious	THC SDB-001 JWH-018	1–10 i.p. 0.3–3 1–10	↓HR ↓HR ↓HR	the effect of THC on HR is shared by another two CB-R agonists (antagonists not used)	[25]
mice	conscious	cannabis THC (10–14%) and CBD (0–2%)	cigarettes via Smoke Inhalation System	↑serum COHb modulation of the proportionality of innate immune-cell populations in the lungs	increase in serum COHb level and modification of immunological system	[148]
mongrel dogs	morphine, chloralose	THC	1 and 2.5 i.v.	↑HR (dose-dependent) ↓RVSW	antagonists not used	[137]
mongrel dogs	pentobarbital	THC	2.5 i.v	↓HR	the maximal THC-induced bradycardia occurred only when both sympathetic and parasympathetic innervation to the heart was intact	[149]
mongrel dogs	pentobarbital	THC	2.5 i.v	HR was constant by electrical pacing; ↓CO, ↓SV, ↓LVP ↓LVEDP, ↓+dp/dt	↓CO mainly due to diminished venous return to the heart and not to impaired contractile force of the myocardium (experiments in which CO was constant by a right heart-bypass procedure)	[150]
cats	chloralose	THC THC	2 i.v. 2 i.c.v.	↓HR ↓HR	bradycardia induced by a central mechanism was diminished by cervical cardiac denervation but not by vagotomy	[151]
cats	pentobarbital	THC	0.2 i.v	↓HR	bradycardia antagonists not used	[152]
mice	isoflurane	THC	0.002 i.p.	↔HR, LVESD, LVEDD, FS	antagonists not used; lack of effect not surprising since a very low dose was chosen	[153]
Wistar rats	urethane	THC	1, 2, 5 i.v.	↓HR	bradycardia due to alteration of efferent vagal activity (blocked by vagotomy and atropine i.v.)	[142]
Sprague Dawley rats	urethane	THC ∆^8^-THC	0.5 i.v. 0.5 i.v.	↓HR ↓HR	bradycardia	[152]
Sprague Dawley rats	pentobarbital	RIM AM251	3 i.v. 3 i.v.	prevention (by RIM but not AM251) of the LPS-induced ↓cardiac contractility (+dp/dt and LVSP) but not of ↑HR	a cardiac receptor distinct from CB_1_-R or CB_2_-R mediates negative inotropy	[154]
Sprague Dawley rats	diethyl ether + urethane	THC THC HU-210 CP WIN-2 JWH-015 AEA	0.03–10 i.v. 30 i.v. 0.003–0.3 i.v. 0.001–0.3 i.v. 0.01–10 i.v. 3–30 i.v. 4 i.v.	↓HR dose-dependent ↑HR ↓HR dose-dependent ↓HR dose-dependent ↓HR dose-dependent ↓HR dose-dependent initial and delayed ↓HR	bradycardia induced by all compounds mediated by CB_1_-Rs (blocked by RIM); in the case of AEA, only the delayed ↓HR was diminished by RIM	[155]
Sprague Dawley rats	pentobarbital + isoflurane	JWH-030	0.1 i.v. 0.5 i.v.	↔ QT interval ↑ QT interval, ↔RR interval	prolongation of the QT interval may be associated with adverse cardiovascular effects in abusers of synthetic cannabinoids	[156]
Wistar rats	chloralose	HU-210 AEA MethAEA ACPA	0.1 i.v. 2.5 i.v. 2.5 i.v. 0.125 i.v.	↓HR, ↔ECG ↓HR, ↑duration QRS ↓HR ↔ ECG ↓HR ↔ ECG	bradycardia mediated by CB_1_-Rs (inhibited by RIM but not by SR144528)	[157]
mice TRPV1^+/+^ TRPV1^−/−^	pentobarbital	AEA	20 i.v.	brief (Phase I) and profound (Phase II) ↓HR, LVSP, LVEDP, +dp/dt, −dp/dt	brief (Phase I) and profound (Phase II) bradycardia and ↓cardiac contractility due to AEA mediated via TRPV1- and CB_1_-Rs, respectively (absent/present in TRPV1^−/−^ and not modified/blocked by RIM, respectively); *basal* LVSP, LVEDP, +dp/dt, −dp/dt and HR did not differ between TRPV1^+/+^ and TRPV1^−/−^	[158]
Wistar rats	urethane	WIN-2 CP	0.03–1 i.v. 0.03–1 i.v.	↓HR and plasma [NA] less pronounced in ventilated than in spontaneously breathing rats	depressive action of CBs depends on the respiratory state of the animals and CB_1_-Rs inhibiting sympathetic and intensifying cardiac vagal tone, since ↓HR and plasma [NA] were diminished by RIM and methylatropine	[159]
	urethane plus pancuronium	WIN-2	0.03–1 i.v.			
mice GPR55^+/+^ GPR55^−/−^	ketamine/ xylazine	deletion of GPR55	-	GPR55^−/−^: *young*: ↑HR, ↔ most other cardiac functions; *mature*: cardiac dysfunction (↑LVESV, ↑LVEDV, ↓EF) and ventricular remodeling (↓LV free wall thickness, ↓heart weight/body weight ratio); *young and mature*: ↓cardiostimulatory responses to α_1_/β_1_-AR agonist dobutamine	GPR55 involved in the control of adrenergic signalling	[87]
mongrel dogs (spinal)	pentobarbital	THC	2.5 i.v	↔ HR; ↔ increases in HR induced by ES or by ISO	THC is devoid of any ganglionic or β-adrenergic blocking properties	[149]
Wistar rats (pithed) ^2^	Pentobarbital ^3^	WIN-2 CP MethAEA WIN-3	0.0005–0.5 i.v. 0.0004–0.4 i.v. 1.1, 3.6 i.v. 0.0005–0.5 i.v.	↔HR, ↓increases in HR induced by ES ^5^ and NIC ↔increases in HR induced by ISO ↔ ES ^5^ increases in HR	↓neurogenic sympathetic neuroeffector transmission in the heart via CB_1_-Rs located prejunctionally on the postganglionic rather than on the preganglionic sympathetic nerve fibers innervating the heart (blocked by RIM and/or AM251) and not on the chromaffin cells of the adrenal medulla (inhibitory effect of MethAEA on NIC-induced increase in HR not modified by AM251)	[40,160]
Wistar rats (pithed, adrenalectomized) ^2^	Pentobarbital ^3^	WIN-2 CP	0.0005–0.5 i.v. 0.0004–0.4 i.v.	↔HR, ↓increases in HR induced by ES ^5^ and NIC		[40]
Wistar rats (pithed) ^2^	pentobarbital or urethane ^3^	CP	0.4 i.v.	↔HR, ↓increases in HR induced by ES ^5^	stronger inhibitory effect of CP on the neurogenic tachycardic response in pentobarbitone- than in urethane-anaesthetized rats	[161]
Wistar rats (pithed) ^3^	Pentobarbital ^2^	WIN-2 CP WIN-3	0.0005–0.5 i.v. 0.0004–0.4 i.v. 0.0005-0.5 i.v.	↔ HR, ↓decreases in HR induced by ES of n. vagus ↔decreases in HR induced by methacholine	presynaptic CB_1_-Rs located on the post- and/or preganglionic cardiac vagal nerve fibers did not modify the vagal bradycardia	[40]
rabbits (pithed) ^4^	pentobarbital	WIN-2 CP	0.005–1.5 i.v. 0.003–1 i.v.	both: ↓increase in HR induced by ES ^5^ WIN-2: ↔increase in HR induced by ISO	↓neurogenic sympathetic and vagal neuroeffector transmission in the heart (via CB_1_-Rs on pre- or postganglionic neurons; blocked by RIM)	[139]
		WIN-2 CP	0.005–0.5 i.v. 0.003–0.3 i.v.	↓ES decreases in HR elicited by ES of n. vagus		
Wistar rats (pithed) ^2^	Urethane ^3^	AEA CP	1 i.v. 0.4 i.v.	↔HR; ↓increases in HR induced by ES ^5^	↓neurogenic sympathetic tachycardia due to the presynaptic CB_1_-R but not GPR18 (blocked by AM251 but not O-1918, respectively)	[162]
Sprague Dawley rats (pithed)	ether	THC	1 i.v.	↔HR; ↔alterations in HR induced by ISO and propranolol	β-adrenoceptors not involved in the cardiac action of THC	[163]
hairless mice	ketamine and xylazine	AEA CBD WIN-2	5 i.p. 5 i.p. 5 i.p.	AEA (unlike CBD and WIN-2) ↓venular thrombus formation in ear venules	↓thrombus formation evoked by AEA was dependent on cyclooxygenase metabolites (it was reduced by INDO i.p.)	[164]

We have focused on cardiac (but not blood pressure) responses induced by CB-R agonists; CB-R antagonists were mentioned only if their cardiac effects were determined independent of CB-R agonists. If not stated otherwise, antagonists did not modify cardiac parameters by themselves. ^1^ If not stated otherwise, doses are given in mg/kg; ^2^ vagotomized and pretreated with atropine 1.5–2 μmol/kg i.p. and pancuronium 0.8 μmol/kg i.v; ^3^ vagotomized and pretreated with propranolol 3 μmol/kg i.v.; ^4^ pretreated with i.v. methylatropine (1 mg/kg bolus plus an infusion of 2 mg/kg/h) and gallamine triethiodide (5 mg/kg) or succinylcholine (1 mg/kg); ^5^ electrical stimulation of preganglionic sympathetic nerves. ↑increase; ↓decrease; ↔no effect; +dp/dt, maximum rates of contraction; −dp/dt, maximum rates of relaxation; [NA], noradrenaline concentration; α_1_/β_1_ AR, α_1_/β_1_-adrenergic receptors; ∆^8^-THC, Δ^8^-tetrahydrocannabinol (previous nomenclature Δ^6^-THC); ACPA, arachidonoylcyclopropylamide; Adr, adrenaline; AEA, anandamide; BP, blood pressure; CB_1_-R_,_ cannabinoid CB_1_ receptor; CB_2,_ cannabinoid CB_2_ receptor; CBD, cannabidiol; CBs, cannabinoids; CO, cardiac output; COHb, carboxyhemoglobin; CP, CP55940; eCBs, endocannabinoids; ECG, electrocardiogram;LEF, ejection fraction; ES, electrically stimulated; FS, fractional shortening; GPR55, G protein-coupled receptor 55; HR, heart rate; i.a., intraarterially; i.c., intracisternally; i.c.v., intracerebroventricularly; i.m., intramuscularly; INDO, indomethacin; i.p., intraperitoneally; ISO, isoprenaline; i.v., intravenously; LPS, lipopolysaccharide; LV, left ventricle; LVEDD, left ventricular end-diastolic diameter; LVEDP, left ventricular end-diastolic pressure; LVEDV, left ventricular end-diastolic volume; LVESD, left ventricular end-systolic diameter; LVESV, left ventricular end-systolic volume; LVID, left ventricular internal diameter; LVP, left ventricular peak; LVSP, left ventricular systolic pressure; MethAEA, methanandamide; n., nerve; NIC, nicotine; RIM, rimonabant; RVSW, right ventricular stroke work; s.c., subcutaneously; SV, stroke volume; THC, Δ^9^-tetrahydrocannabinol (previous nomenclature Δ^1^-THC); TRPV1, transient receptor-potential cation-channel subfamily V member 1; WIN-2, WIN55212-2; WIN-3, WIN55212-3.

## Data Availability

Not applicable.
